# Supplementary material for: Light-dependent N-end rule-mediated disruption of protein function in Saccharomyces cerevisiae and Drosophila melanogaster
Source: PLoS Genet. 2021 May 17;17(5):e1009544. doi: 10.1371/journal.pgen.1009544 (PMC8158876; doi:10.1371/journal.pgen.1009544)
Supplement: Attachment — Submitted filename: Responses to Reviewers comments on Stevens et al.docx [file pgen.1009544.s009.docx]

Response to reviewers’ comments:

To begin with, we were very pleased to receive the positive reviews from the two reviewers. We would like to thank them for their thorough reviews and for their constructive comments, all of which we have addressed as outlined below.

Reviewer #1

“line 199. The authors cite for the first time the use of the yeast *UBR1 ura3* mutant strain. It would be helpful to add a sentence explaining the overall characteristics of that strain, which is used several times throughout the manuscript. This would avoid having to report back to the original paper for basic information. Alternatively, the same type of information could be added to the methods section.”

In the original submission of the manuscript, we included the complete genotypes of both the *UBR1* *ura3* strain, YPH500, and its *ubr1Δ* derivative, JD15, in the **Materials and Methods** section, under the heading “**Yeast and *Drosophila* strains and Maintenance**.” In addition, we now include additional explanatory sentences regarding the strains used in the Results section immediately following line 199, when they are first mentioned in the text.

line 315. The authors examine whether PND-dependent degradation occurs post-translationally or by degradation of nascent peptides, the latter process limiting the use of the tool for the study of loss-of-function phenotypes. They do so by growing PND-HA-Cdc28 expressing cells in the presence of cycloheximide and find that protein degradation in the presence of light is still observed, suggesting a post-translational mechanism. While this seems true for *cdc28*, different effects could, in principle, hold for other genes and must be acknowledged. That is, is it possible that the test performed in the presence of cycloheximide only rules out a co-translational effect for Cdc28p but not necessarily for other genes? To discuss this possibility prepares the reader for limitations they might encounter should they consider using this tool for their own protein of interest.

Reviewer #1 brings up a good point. Other proteins may exhibit different properties under the control of the PND, the B-LID, and the psd, and we have emphasized this point in the manuscript. I would make the point that our Western blot analysis does not prove that PND-HA-Cdc-28 does not undergo co-translational degradation. In fact, we suspect that some of the protein does undergo co-translational degradation. Rather, the Western blot analysis, as well as the observation that many PND-HA-Cdc-28- and PND-HA-Ura3p-expressing yeast arrest as single cells indicates that full-length mature protein undergoes post-translational degradation, and does not perdure for a long time (or multiple cell cycles) before undergoing degradation, which would undermine the use of this technology for phenotypic analysis. Nevertheless, we agree that some PND-tagged mature proteins might not undergo rapid degradation and that this must be tested empirically on a protein-by-protein basis. Accordingly, we have added some additional text outlining these issues in the final paragraph of the section describing the studies of PND-HA-Cdc-28.

For their *Drosophila* studies, Stevens et al use a dominant negative form of the IkB inhibitor Cactus that is insensitive to Toll-induced degradation (cactDN), generating a dorsalizing phenotype. If degradation is effective, the embryonic phenotype is reverted by illumination, a smart way to study the utility of the degrons to access protein function in vivo. They generate several insertion lines expressing CactDN-degrons and cite that they have some line-to-line variability due to different expression levels (line 407). This generates diversity in the number and severity of dorsalized embryos grown in the dark. Since their table containing results on the viability of these embryos gathers the different lines, I am curious about how this variability in expression levels may alter the effectiveness of the degron for the study of protein function. It would be interesting if they could add this information.

In response to this comment, we have included information about line-to-line variability in cuticular phenotypes generated by the three degron-tagged CactDN constructs. The results show that for the three degrons tested, the majority of the individual transgenic lines show the same relative pattern of severity of dorsalized cuticular phenotypes as does the combined total for the corresponding degron.

The authors cite (line 541):
"our studies of the ability of the cactDN degron to control the nuclear accumulation of Dorsal-GFP indicate that ...PND and B-LID are capable of mediating temporally-specific elimination of Cact within the blastoderm embryo"
Temporal control is certainly a strong characteristic shown for the degrons above and should be very useful for developmental studies. Since they have used the same tag for several yeast proteins, I wonder whether they could compare the level of temporal control attained with those proteins as well. If they could explore a bit more this issue it would be useful in giving a sense of the generality of this phenomenon.

Our western blot analysis of PND-HA-Cdc28p demonstrated directly that loss of that protein is much more rapid in cells under illumination than it is in cells grown in darkness. We observed that most of the protein is lost within 15 minutes of illumination so the half-life of the protein must be less than 15 minutes. We chose PND-HA-Cdc28p for these studies because it is chromosomally integrated, with expression levels likely to be fairly uniform from cell-to-cell. PND-HA-Ura3p and PND-yEmRFP were introduced into cells on extrachromosomal plasmid vectors and although those are purported to be present at a single copy per cell, it is unclear whether those vectors are as stably inherited or expressed as the same levels as PND-HA-Cdc28, which could complicate the direct comparisons of the rates of loss of the three proteins.

In addition, the rationale underlying these studies was that observable **phenotypes** could rapidly result from light-mediated degradation of degron-tagged proteins-of-interest. As the amount of a particular protein present in a cell that is required to support wild-type function will differ among proteins, it is the time that is necessary to (achieve the loss of protein required to) detect a loss-of-function phenotype, not the rate of protein degradation, that is important in determining the utility of this approach for a particular protein. In the case of PND-HA-Ura3p and PND-HA-Cdc28p, that interval appears to be within a single cell cycle. As GFP and its mutant derivatives (such as yEmRFP) are very stable proteins, we suspect that despite our ability to detect a loss of PND-yEmRFP after 24 or 48 hours of growth, it may not be possible to see a substantial loss of PND-yEmRFP-dependent fluorescence from single cells within a one cell cycle interval.

Finally, as noted by this reviewer (re: line 315) and as stated in the manuscript several times (line 459, lines 595-598, line 603), different proteins exhibit different intrinsic stabilities that are likely to influence the rates of degradation mediated by the light-inducible degrons and the time that it will take for light-dependent loss of protein to generate a detectable phenotype. Taken together, these and other observation have led to our conclusion that for a given protein-of-interest, it must be determine empirically whether any or all of the available light-dependent degrons will be useful for its study. As we have been able to show a clear light-dependent loss of protein function for 4 out of the 4 proteins that we have tagged with the PND, with 3 of those proteins (Ura3p, Cdc28p, CactDN) exhibiting the expected biologically-relevant phenotype, it is not clear to us that performing a more temporally-precise analysis of the rate of loss of PND-HA-Ura3p and PND-yEmRFP is necessary to support the conclusions of our paper.

Discussion, line 602: The authors consider differences in intrinsic protein stability an important property to take into account when devising a degron to study loss-of-function phenotypes for a protein of interest. In the same line of reasoning, could they discuss whether and how expression levels would impact degron effectiveness?
This information may be useful especially considering the advent of CRISPR technology. Optogenetic tags that were tested with expression constructs by Stevens et al may be useful to tag endogenous genes, as considered in the Discussion. However, one limitation that can be foreseen is the expression levels obtained in tagging endogenous genes as compared to overexpression constructs. Do the authors have information on the expression levels of their tagged constructs versus endogenous expression? The western blot in Fig 8 shows slightly higher levels of Cact-B-LID as compared to endogenous Cact levels. On the other hand, in their paper using a Dl-degron they generate much higher levels of construct expression compared to endogenous. Do they think this is an important parameter to be considered for different proteins?

Reviewer #1 makes an excellent point here, with regard to the influence of expression levels and the use of gene replacement by degron-tagged constructs. To answer the reviewer’s question, aside from CactDN-B-LID, we have not compared the levels of degron-tagged proteins with that of the endogenous proteins. In the case of CactDN-B-LID versus endogenous Cactus, it is not clear that that comparison is very informative, as CactDN-B-LID is not subject to Toll signal-dependent degradation but is subject to B-LID-mediated degradation, while endogenous Cactus is subject to Toll signal-dependent degradation but not to B-LID-mediated degradation. The same issue is at play for the PND-HA-CactDN, however that is further complicated by the fact that the monoclonal anti-Cactus antibody that we used to detect CactDN-B-LID does not recognize PND-HA-CactDN and endogenous Cactus is not detected by the anti-HA antibody with which we performed western blot analysis of PND-HA-CactDN. We did not analyze CactDN-psd on western blots as there was little evidence to suggest that the psd was eliciting light-dependent degradation. In the case of the yeast studies, we were not able to identify sources of antibodies directed against endogenous Ura3p or Cdc28p and therefore used anti-HA for our analysis of PND-HA-Cdc28p and the corresponding endogenous proteins would not be expected to be detected by the anti-HA antibody. In the case of PND-yEmRFP, there is no endogenous gene with which to compare it.

However, the question of expression levels of degron-tagged proteins versus their corresponding endogenous genes is an important one. Our decision to use conventional P-element based transformation in order to introduce the pUASp-based vectors carrying the three degron-tagged versions of CactDN was directed by the fact that sites of insertion can affect the level of expression of transgenic insertions. As we were uncertain of the amount of transgenic protein that would be required to hit the “sweet spot”, the optimal level of protein for which dark incubation would lead to a strong dorsalized phenotype while illumination would yield wild-type embryos, we decided to use an approach that could generate insertion lines with different levels of transgene expression. In the case of CactDN-psd, we did not generate any transgenic lines that hit this optimal concentration. In the case of PND-HA-CactDN and CactDN-B-LID, we did obtain some transgenic lines that fell within this range. The use of CRISPR/Cas9 to generate insertions of degron tagged genes has the significant advantage of leading to the correct spatial and temporal pattern of expression of proteins-of-interest. However, it is far from clear that this approach will generate protein expression levels that are equivalent to that of the endogenous genes for a simple reason. In all cases, this involve adding a degradation signal that is not present in the endogenous protein. Even in darkness, the PND, B-LID and the psd are degradation signals that are likely to, at least weakly, destabilize the tagged proteins in comparison to their endogenous counterparts. Reviewer #1’s comment that Irizarry et al., 2020 shows that Dorsal-B-LID is present at higher concentrations than endogenous Dorsal is not correct. In fact, the opposite is true. Dorsal-B-LID is present at lower levels than endogenous Dorsal and is more subject to degradation in the dark than endogenous Dorsal (See Irizarry et al., 2020, Figure 1C versus 1G, 1E, and Figure S1B-D). The interpretation of these results was that while degradation of Dorsal-B-LID is efficient with blue-light exposure, B-LID-mediated degradation, while much less efficient, is leaky, with some degradation occurring even in the dark. Accordingly, one must be cautious in choosing to study protein function following replacement of an endogenous gene by its degron-tagged alternate. There may be cases in which overexpression of the degron-tagged version in the background of a corresponding mutant in the endogenous gene is more useful. We have added some comments regarding these issues in the discussion of this revised version of the manuscript.

Methods and legends:
About the GAL4 drivers used in the *Drosophila* experiments: in line 360 the authors cite the use of the *nanos-GAL4* driver (Table 1). Is this used for Fig 7A also?
In Fig 7B and Table 2 it is also unclear which GAL4 is used. Since nothing is said in legends, one assumes it is the same GAL4 driver as the previous figures. However, in Methods they state that for embryo illumination they use either nanos or mat GAL4 drivers (line 929) for viability and cuticle phenotypes, but do not specify in each experiment which GAL4 driver was used. Please specify. For western blot experiments I could not find information as to which GAL4 driver was used.

The *nanos-Gal4:VP16* transcriptional driver element was used for the analysis of hatch rates and cuticular pattern elements and for western blot analysis. The *mat-α4-tub-Gal4:VP16* driver element was utilized for the live imaging of Dorsal-GFP in the background of embryos expressing the various CactusDN constructs. This information is now stated explicitly in the Materials and Methods.

Reviewer #2: In this manuscript, the authors describe the development of photo-N-degron (PND), an N-terminal blue light-inducible degron, for the induction of loss-of-function phenotypes in yeast and in *Drosophila*. They tested the feasibility of PND both by phenotypic and biochemical (western blots) analysis. In the first part of the manuscript, the authors describe the use of PND in three examples in yeast. Here, they also show that the degradation of PND is dependent on the N-end rule degradation pathway. In the second part, the authors tested the functionality of PND in a multi-cellular organism, Drosophila. Specifically, they make a fusion of PND with Cactus[DN] which cases a dominantly dorsalizing phenotype in *Drosophila* embryos. Both in the yeast and *Drosophila* assays, the authors demonstrate that PND can be used for blue light-induced protein degradation. In the *Drosophila* part, the authors also compared the PND to two other degrons, PSD and B-LID, which are carboxy-terminal light-induced degrons. While the Cact-PSD fusion does not appear to respond to blue-light illumination, the Cact-B-Lid fusion responds very well, maybe even better than PND, to blue light. The authors also examine in great detail the effect of the Cact[DN] fusion proteins on the nuclear localization of Dorsal in light and dark conditions.

Overall, this is an interesting study which introduces an N-terminal blue light-inducible degron for optogenetic studies of protein function *in vivo*. It increases the toolbox for these kinds of studies and increases the flexibility of researchers to either target proteins at the N- or C-terminus. The experimental procedures used in this study are well explained and the conclusions are supported by the data. I have only a few minor concerns/questions to further improve the manuscript.

1. My major concern is the length of this manuscript. It is way too long and should be shortened. There are many redundant parts where the authors repeat themselves over and over. Figure 7 and Tables 1 and 2 contain the same content. Either the tables or the figure could be removed. The Discussion is very good, but also too long.

In response to reviewer #2’s comment, we have shortened the manuscript. We have removed Figure 7, as suggested, and its associated legend. We also have removed S9 movie as it was not essential in supporting the conclusions of the paper. As noted by the reviewer, there were some repetitive passages in the paper. In most of those cases, the Discussion section reiterated information that had be presented in either the Introduction or the Results sections. Accordingly, we edited and reorganized the Discussion to remove passages that repeated information that had been provided elsewhere and this shortened the length of the Discussion and of the manuscript overall.

2. In Figure 2, it wasn't clear to me why a *ura3* mutant strain was used, at least this was mentioned in the text. In the actual figure and legend, *ura3* was not mentioned. Please clarify.

In the experiments displayed in Figure 2, the same strains of yeast, YPH500 and JD15, were used as had been used in the studies of the Ubi-R-phLOV2(PND)-HA-Ura3p protein, in which the *ura3* mutant background was important. In the interest of continuity, we used the same nomenclature for that strain in the description of the experiments outlined in Figure 2. However, as the *ura3* mutation in YPH500/JD15 is immaterial to the experiments shown in Figure 2, I have deleted the *ura3* designation from that segment of the text.

3. In Figure 7, the authors use D4 as an additional class of dorsalized embryos, but don't explain it in the text. In line 395, the introduce UH as abbreviation for unhatched embryos, but don't use it the figure. Is it possible that D4 corresponds to UH?

UH (for unhatched) is the same as D4. As Figure 7 was the only location in which the designation D4 was used, and since Figure 7 has been omitted in the interest of shortening the manuscript, this issue has been resolved.
